# Supplementary material for: Measurements of heterogeneity in proteomics analysis of the nanoparticle protein corona across core facilities
Source: Nat Commun. 2022 Nov 3;13:6610. doi: 10.1038/s41467-022-34438-8 (PMC9633814; doi:10.1038/s41467-022-34438-8)
Supplement: Supplementary file 1 — Supplementary Information [file 41467_2022_34438_MOESM1_ESM.pdf]

Supplementary Information

Measurements of heterogeneity in proteomics analysis of nanoparticle protein corona across core facilities

Ali Akbar Ashkarran[#1], Hassan Gharibi[#2], Elizabeth Voke[3], Markita P. Landry[3,4,5,6], Amir Ata Saei[2,7*§], and Morteza Mahmoudi[1*]

[1]Department of Radiology and Precision Health Program, Michigan State University, East Lansing, MI, USA

[2]Division of Physiological Chemistry I, Department of Medical Biochemistry and Biophysics, Karolinska Institutet, Stockholm, Sweden

[3]Department of Chemical and Biomolecular Engineering, University of California, Berkeley, Berkeley, CA, USA

[4]Innovative Genomics Institute, Berkeley, CA, USA

[5]California Institute for Quantitative Biosciences, University of California, Berkeley, Berkeley, CA, USA

[6]Chan Zuckerberg Biohub, San Francisco, CA, USA

Department of Cell Biology, Harvard Medical School, Boston, MA, USA

[#]Equal contribution

[§]Current address: Biozentrum, University of Basel, 4056 Basel, Switzerland

*Corresponding author: (AAS) amirata.saei.dibavar@ki.se; (MM) mahmou22@msu.edu

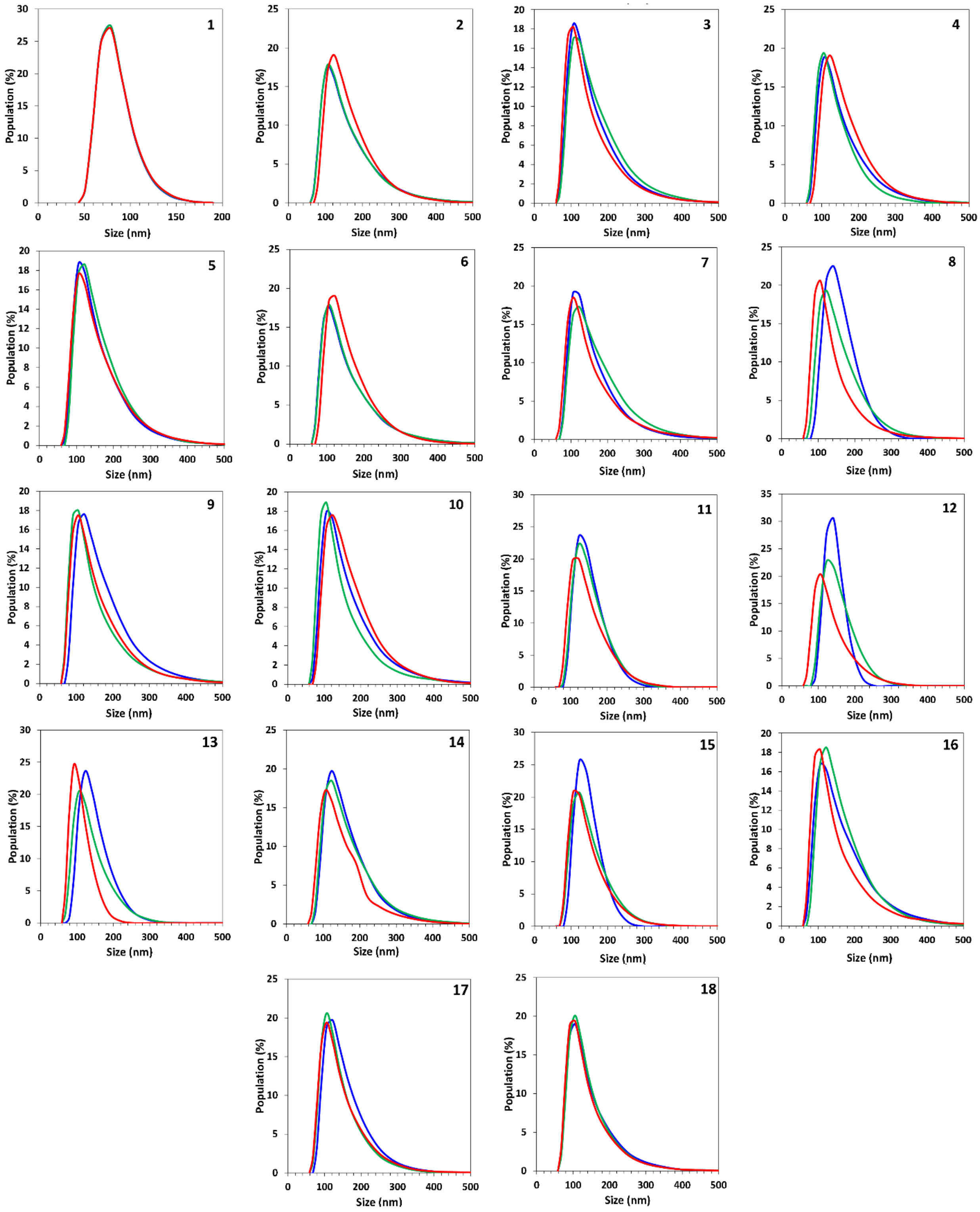

Supplementary Fig. 1: Characterization of different NP batches. Size distribution and the corresponding replicates of (1) bare and (2 to 18) protein corona coated NPs of 17 prepared samples. The results are representative of 3 independent analyses.

Supplementary Table 1: Average size, polydispersity index (PDI), zeta potential, and the corresponding standard deviation values of protein corona coated NPs in 17 prepared samples (all measurements are repeated three times and the corresponding averages are reported).

Sample	Size (nm)	SD (nm)	PDI	Zeta potential (mv)	SD (mv)
Bare NPs	78.8	0	0.028	-31.6	0.3
1	111.3	9.6	0.261	-10.2	0.7
2	108.1	4.2	0.231	-9.6	0.5
3	116.8	9.6	0.270	-9.9	0.4
4	111.3	9.6	0.219	-10.2	0.4
5	111.3	9.6	0.229	-10.4	0.5
6	113.7	8.4	0.177	-9.8	0.1
7	123.3	18.1	0.260	-10.2	0.2
8	111.3	9.6	0.199	-10.3	0.1
9	111.3	9.6	0.194	-10.2	0.5
10	119.3	5.4	0.353	-9.7	0.3
11	123.3	18.1	0.315	-10.1	0.2
12	116.8	9.6	0.299	-10.0	0.5
13	116.8	9.6	0.192	-10.3	0.2
14	116.8	9.6	0.283	-10.1	0.3
15	113.7	8.4	0.197	-9.9	0.3
16	111.3	9.6	0.226	-10.0	0.3
17	111.3	9.6	0.195	-10.2	0.6

Supplementary Table 2: Detail of statistical analysis (i.e., single factor ANOVA) on the size of the protein corona coated NPs and the corresponding averages and *P* values.

Groups	Count	Sum	Average	Variance
1	3	333.8	111.27	92.96
2	3	324.4	108.13	17.76
3	3	350.5	116.83	92.96
4	3	333.8	111.27	92.96
5	3	333.8	111.27	92.96
6	3	341.1	113.70	70.09
7	3	369.9	123.30	326.41
8	3	333.8	111.27	92.96
9	3	333.8	111.27	92.96
10	3	357.8	119.27	29.45
11	3	369.9	123.30	326.41
12	3	350.5	116.83	92.96
13	3	350.5	116.83	92.96
14	3	350.5	116.83	92.96
15	3	341.1	113.70	70.09
16	3	333.8	111.27	92.96
17	3	333.8	111.27	92.96

ANOVA

Source of Variation	SS	df	MS	F	*P* value	F crit
Between Groups	942.91	16	58.93	0.54	0.91	1.95
Within Groups	3725.63	34	109.58			
Total	4668.54	50				

Supplementary Table 3: Details of statistical analyses (i.e., single factor ANOVA) on the zeta potential of the protein corona coated NPs and the corresponding averages and *P* values.

Groups	Count	Sum	Average	Variance
1	3	-28.82	-9.61	0.23
2	3	-30.39	-10.13	0.75
3	3	-29.74	-9.91	0.17
4	3	-30.62	-10.21	0.18
5	3	-31.14	-10.38	0.28
6	3	-29.39	-9.80	0.01
7	3	-30.49	-10.16	0.02
8	3	-30.9	-10.30	0.01
9	3	-30.55	-10.18	0.22
10	3	-29.2	-9.73	0.12
11	3	-30.43	-10.14	0.06
12	3	-29.93	-9.98	0.21
13	3	-30.9	-10.30	0.03
14	3	-30.35	-10.12	0.11
15	3	-29.59	-9.86	0.10
16	3	-30.14	-10.05	0.09
17	3	-30.67	-10.22	0.32

ANOVA

Source of Variation	SS	df	MS	F	*P* value	F crit
Between Groups	2.27	16	0.14	0.82	0.65	1.95
Within Groups	5.84	34	0.17			
Total	8.10	50				

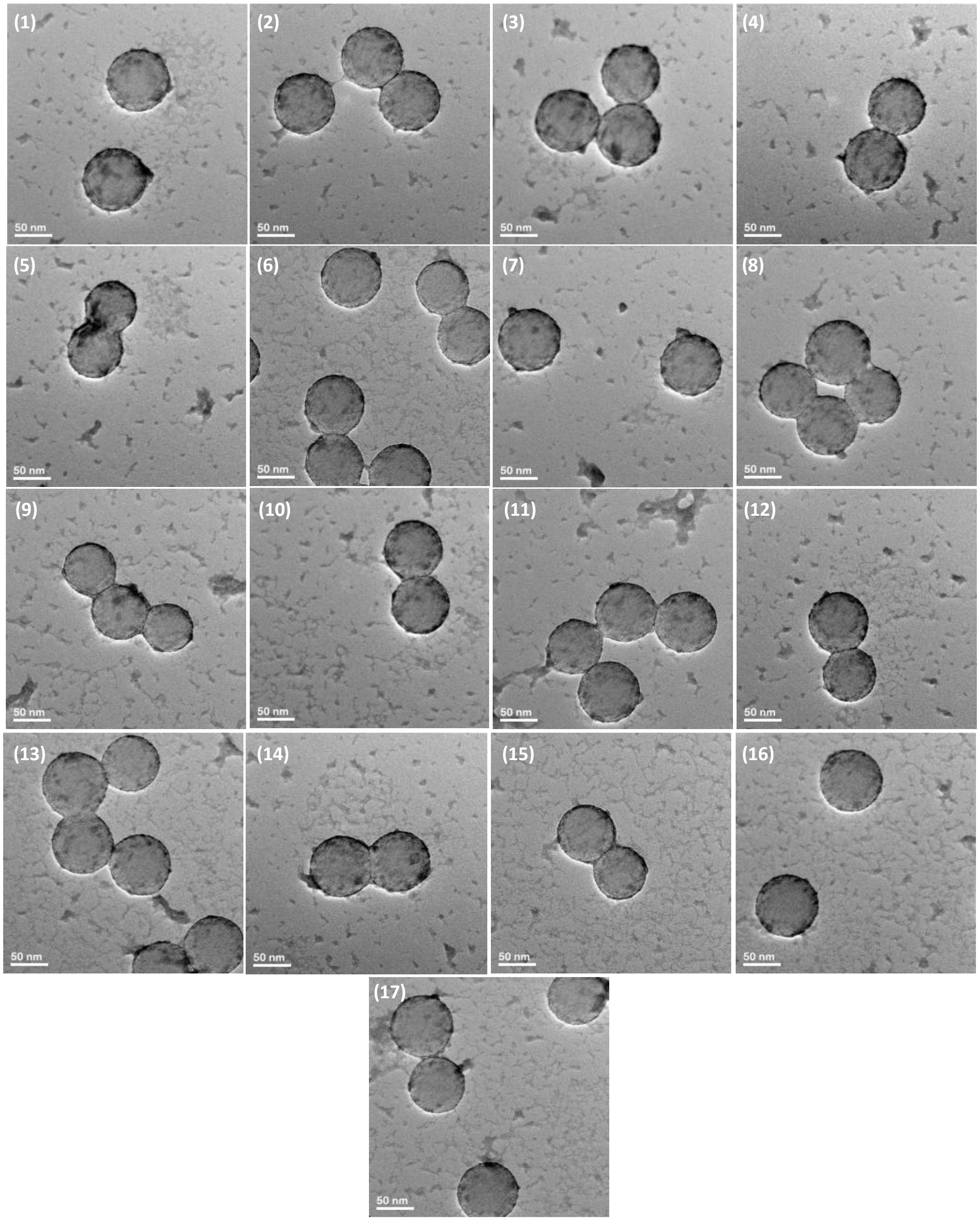

Supplementary Fig. 2: TEM images of protein corona coated PSNPs (1 to 17). After preparation of 17 aliquots, 20 µl of the solution from each aliquot was removed, stained and characterized by TEM analysis. The results are representative of 3 independent analyses.

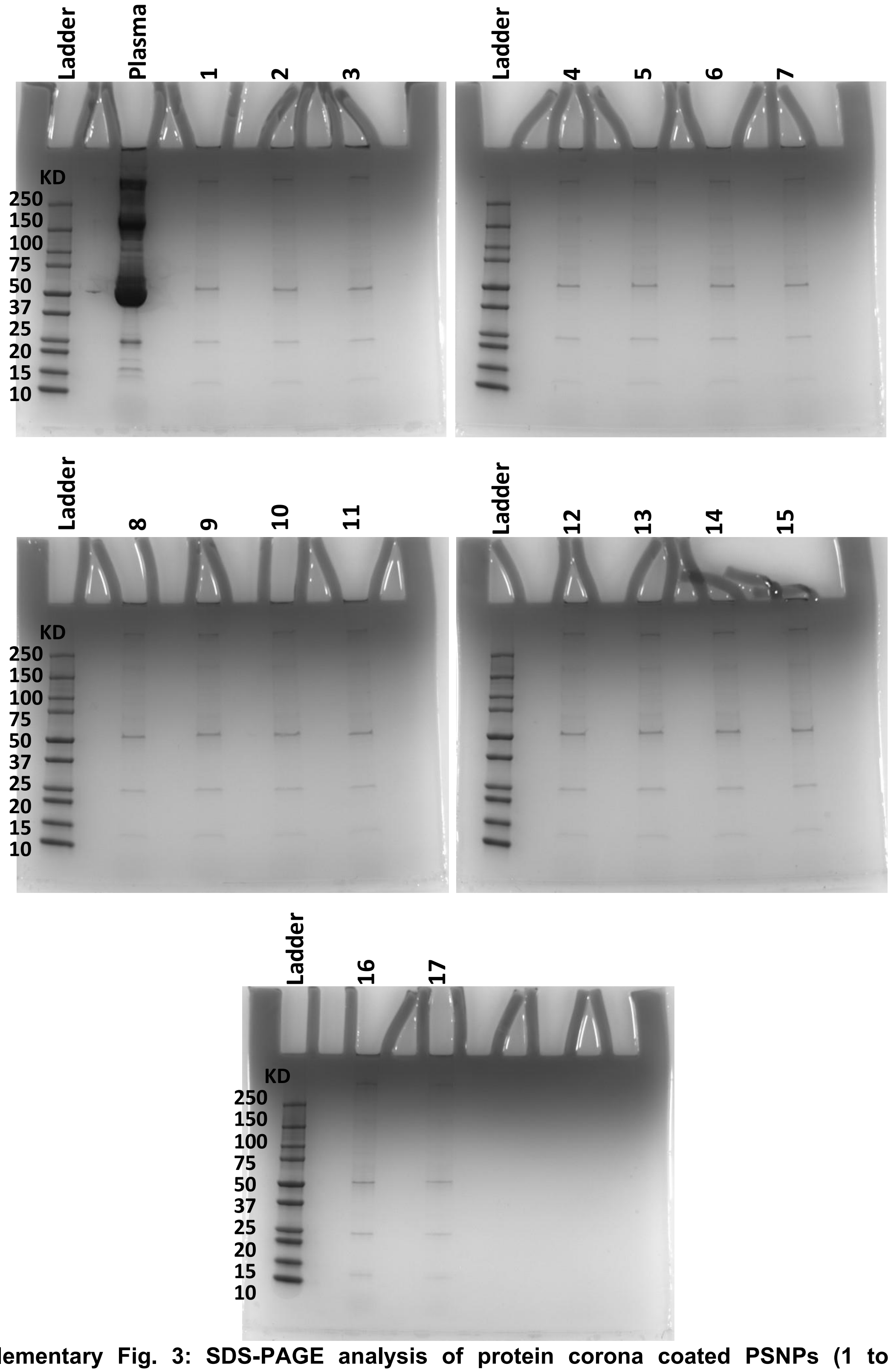

Supplementary Fig. 3: SDS-PAGE analysis of protein corona coated PSNPs (1 to 17). After preparation of 17 aliquot samples, 20 µl of the solution from each aliquot were removed and used for SDS-PAGE analysis. The results are representative of 3 independent analyses.

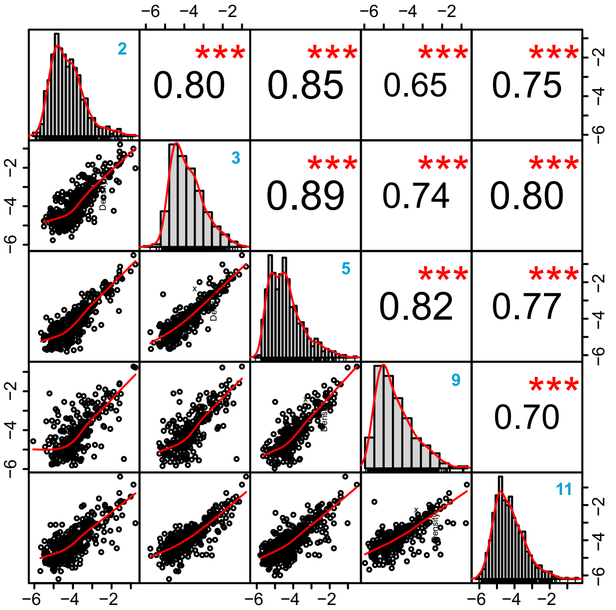

Supplementary Fig. 4: Pearson correlation of protein intensities for 151 proteins shared between 5 centers that outperform the others with regards to number of quantified proteins and peptides, as well as median CVs and sequence coverage. The correlation coefficient is given in black text and core codes are given in bold blue text on the top right corners of the boxes. *** denote a significance below *P* value of 0.001 (derived from Pearson correlation analysis)

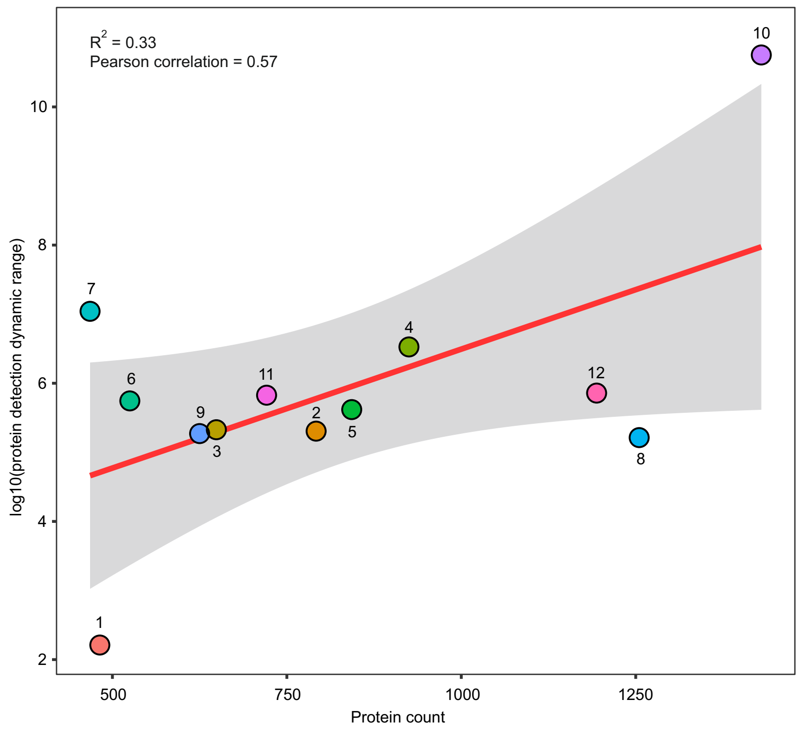

Supplementary Fig. 5: Association of protein detection power (dynamic range) with the number of quantified proteins among the 12 cores. Dynamic range is defined as the ratio of most intense protein divided by least intense protein in data from each core. The red line shows the linear regression of the data points, and the grey area represents the standard error from the regression line at 95% confidence level.

Supplementary Table 4: Comparison of the protocols, LC and MS systems, method durations, and other important parameters between all the cores.

Core	Digestion	Digestion mode	LC system	MS system	Data acquisition mode	Gradient duration	Search engine	Database	Variable modifications and other search parameters	FDR	No. Proteins	No. Peptides	Median CV (%)	Median sequence coverage
**1**	Trypsin overnight at 37 °C	On-bead digestion	Thermo Dionex Ultimate 3000RSLCnano	Fusion Lumos	DDA	NR	MSFragger	SwissProtKB	NR*	<1% at protein and peptide levels	481	3939	32.04	NR
**2**	Trypsin overnight at 37 °C	On-bead digestion	Thermo Dionex Ultimate 3000RSLCnano	Eclipse	DDA	60 min	Proteome Discoverer 2.4	UniProt	M oxidation, acetylation and M loss on protein N-termini	<1% for high confidence and <5% for medium confidence	792	7565	6.62	18
**3**	Trypsin overnight at 37 °C	In-solution digestion	Thermo Dionex Ultimate 3000RSLCnano	Fusion	DDA	90 min	Proteome Discoverer 2.4	NCBI	M oxidation and N/Q deamidation; acetylation, M loss and M loss plus acetylation of protein N-termini	<1% at the peptide level	649	3917	3.08	13
**4**	Trypsin overnight at 37 °C	On-bead digestion	NR	Velos Elite	DDA	NR	NR	NR	NR	Between 1-2% at peptide level	926	NR	13.48	NR
**5**	Trypsin overnight at 37 °C	On-bead digestion	NR	Fusion Lumos	DDA	85 min	Protein Discoverer v.2.4	UniProt	M oxidation, N/Q deamidation, STY phosphorylation and acetylation of protein N-termini, up to 3 missed cleavages were allowed.	<5% at protein and peptide levels	843	5907	8.81	11
**6**	Trypsin according to SOP	On-bead digestion	Bruker nanoElute system	Bruker timsTOF-PRO	Data-dependent PASEF mode	55 min	PEAKS-XPro server	UniProt	M oxidation and N/Q deamidation, semi-specific search	<10% at the peptide level	525	7299	9.44	19
**7**	Promega trypsin or chymotrypsin overnight at 37 °C	On-bead digestion	Waters NanoAcquity	Elite	DDA	73.5 min	PEAKS Studio 10plus	NCBI	NR	<0.5% at protein and peptide levels	468	3345	8.06	9
**8**	Trypsin/Lys-C overnight	On-bead digestion	Waters NanoAcquity	Q Exactive HF-X	DDA	Total gradient of 80 min	Byonic v4.2.4	UniProt	M, H, and W oxidation, dioxidation on M and W, N/Q deamidation, and acetylation on protein N-termini, semi-specific search	<1% at the protein level	1255	5275	12.01	8.3
**9**	Pierce Trypsin/Lys-C Protease mixture overnight at 37 °C	In-solution digestion	Thermo Dionex Ultimate 3000RSLCnano	Fusion Lumos	DDA	60 min	Proteome Discoverer 2.4	SwissProt	M oxidation and acetylation of protein N-termini	<1% at the peptide level; <1% for high confidence	625	3367	6.15	9

										and <5% for medium confidence at protein level				
**10**	Trypsin overnight at 37 °C	On-bead digestion	Thermo Dionex Ultimate 3000RS LCnano	Fusion Lumos	DDA	90 min	Peaks Studio 8.5	NR	NR	NR	1430	6959	21.91	4
**11**	Trypsin overnight at 37 °C	In-gel digestion	NR	Eclipse	DDA	NR	Proteome Discoverer 2.4	UniProt	M oxidation and N/Q deamidation	<1% at the peptide level	721	4768	4.19	14
**12**	Trypsin for 1 h at 47 °C followed by 3 hours at 37 °C	On-bead digestion	Thermo Easy-nLC 1000	Fusion	DDA	NR	Proteome Discoverer 2.4	UniProt	M oxidation and N/Q deamidation	<1%	1194	NR	13.57	6
**13**	Trypsin for 30 min at 55 °C	On-bead digestion	Thermo Dionex Ultimate 3000RS LCnano	Fusion	DDA	90 min	Mascot 2.8	Swissprot	M oxidation	NR	498	3545	NR	NR
**14**	Trypsin/lysC rapid digestion kit from Promega for 70 °C for 1 h	On-bead digestion	Thermo Dionex Ultimate 3000RS LCnano	Q Exactive HF	DDA	120 min	Proteome Discoverer 2.4.0.305	UniProt	M oxidation and N/Q deamidation; acetylation, M loss and M loss plus acetylation on protein N-termini	<5% at the peptide level and 1% at the protein level	336	1539	7.13	10
**15**	Trypsin/Lys-C mixture using the manufacturer protocol	On-bead digestion	Thermo Thermo Dionex Ultimate 3000RS LCnano	Eclipse	DDA	100 min	Proteome Discoverer 2.2.0.388	UniProt	M oxidation, acetylation of the N-terminus and S phosphorylation	<5% at the peptide and protein levels	364	2591	17.81	10.89
**16**	Trypsin overnight at 37 °C	On-bead digestion	EASYnLC 1000	Q-Exactive	DDA	84 min	Mascot Distiller, v2.7.0	UniProt	M oxidation	<1%	235	1058	38.05	5.09
**17**	Trypsin using the manufacturer's protocol	In-gel digestion	Thermo Dionex Ultimate 3000RS LCnano	Elite	DDA	60 min	Mascot	NCBI	M oxidation, up to 5 missed cleavages were allowed.	NR	1339	1505	NR	NR

*NR=not reported.

Supplementary Methods

The following sections describe the materials/methods, details of sample preparation and LC-MS/MS analysis provided by each individual proteomic center and reported here as received (with some minor changes for consistency of units, etc.). The 17 different centers that contributed to this study include Case Western Reserve University (Link), University of Cincinnati (Link), Cornell University (Link), Harvard University (Link), University of Kansas Medical School (Link), University of Missouri (Link), Massachusetts Institute of Technology (Link), Stanford University (Link), University of Tennessee (Link), University of California San Diego (Link), University of Nebraska–Lincoln (Link), Wayne State University (Link), University of Illinois (Link), University of Florida (Link), University of Nevada Reno (Link), Michigan State University (Link), and University of Texas at San Antonio (Link) blindly numbered from 1 to 17.

Center #1: To digest bead-bound proteins, a 10 µL (10 ng/µL) aliquot of trypsin in 100 mM ammonium bicarbonate was added directly onto the washed beads, using an enzyme-to-protein ratio of 1:100 (wt/wt). The samples were vortexed for 15 s every 2–3 min for the first 15 min to ensure that the beads are evenly suspended in the protease solution. Digestion was continued overnight at 37 °C in an oven incubator without further agitation of the beads. After the overnight digestion, a second 10 µL aliquot of protease was added to each sample, and samples were digested for an additional 4 h at 37 °C. The tubes were placed on the magnetic rack and the supernatant was removed. The digested samples were diluted with 100% (vol/vol) formic acid to give a final concentration of 5% formic acid (vol/vol). The digests were cleaned using a C18 ultra micro spin column according to manufacturer's instructions. These samples were dried in a SpeedVac and reconstituted in 1% acetic acid.

The LC-MS system was a Dionex Ultimate 3000 nano-flow HPLC interfacing with a ThermoScientific Fusion Lumos mass spectrometer system. The HPLC system used an Acclaim PepMap 100 precolum (75 µm x 2 cm, C18, 3 µm, 100 A) followed by an Acclaim PepMap RSLC analytical column (75 µm x 15 cm, C18, 2 µm, 100 A). 5 µL volumes of the extract were injected and the peptides eluted from the column by an acetonitrile/0.1% formic acid gradient at a flow rate of 0.3 µL/min were introduced into the source of the mass spectrometer online. The micro-electrospray ion source is operated at 2.5 kV. The digest was analyzed using the data dependent multitask capability of the instrument acquiring full scan mass spectra to determine peptide molecular weights and product ion spectra to determine amino acid sequence in successive instrument scans. The data were searched against the human SwissProtKB protein database with the program Sequest and MSFragger. Protein and peptide validations were performed with the program Scaffold to <1% FDR at the protein and peptide level.

Center #2: 125 µL of the single sample provided in PBS was removed into a separate tube and digested with 50 ng of Promega porcine trypsin overnight at 37 °C. The reaction with stopped with 5% formic acid to a final concentration of 0.5%. The

extracted peptides were dried via SpeedVac, reconstituted in 0.1% formic acid followed by desalting and concentration using the C18 stage tip method.[1] The eluant from the stage tip was dried, reconstituted in 20 µL of 0.1% formic acid and injected as 3 technical replicates of 5.5 µL each for nanoLC-MS/MS analysis. Mass spectrometry data were collected on an Orbitrap Eclipse mass spectrometer coupled to a Dionex Ultimate 3000 RSLCnano system (ThermoFisher Scientific). Samples were injected onto a 5 mm nanoviper µ-Precolumn (i.d. 300 µm, C18 PepMap 100, 5.0 µm, 100 Å) from ThermoFisher Scientific at 5 µl/min in formic acid/H_2O 0.1/99.9 (v/v) for 5 min to desalt and concentrate the samples. For the chromatographic separation of peptides, the trap-column was switched to align with the EASY-Spray column PepMap RSLC C18 with a 150 mm column (i.d. 75 µm, C18, 3.0 µm, 100 Å). The peptides were eluted using a variable mobile phase (MP) gradient from 98% phase A (Formic acid/H_2O 0.1/99.9, v/v) to 32% phase B (Formic Acid/Acetonitrile 0.1/99.9, v/v) for 60 min at 300 nL/min. MS1 data were collected in the Orbitrap (120,000 resolution; maximum injection time 50 ms; AGC 4×10^5). Charge states between 2 and 6 were required for MS2 analysis, and a 20 s dynamic exclusion window was used. Cycle time was set at 2.5 s. MS2 scans were performed in the ion trap with HCD fragmentation (isolation window 0.8 Da; NCE 30%; maximum injection time 40 ms; AGC 5×10^4). The data was recorded using Xcalibur 4.3 software (ThermoScientific).

Protein identification and peptide intensities were generated using Proteome Discoverer 2.4 (ThermoScientific) searched against the UniProt human database (UP000005640 downloaded on 12/11/2020) with the Sequest HT search algorithm and using a modified LFQ standard processing and consensus workflows. In the processing workflow, the mass recalibration node (spectrum files RC) along with the standard spectrum selector, minora feature detector, Sequest HT and Percolator nodes were used. The precursor detector node was used to help minimize chimeric spectra. The precursor mass tolerance was 10 ppm and the fragment mass tolerance was set to 0.02 Da, with 2 missed trypsin cleavages and variable peptide modifications including oxidized methionine and N-terminal protein modifications of acetyl and methionine loss. FDR tolerances in the Percolator node were set to 0.01 for high confidence and 0.05 for medium confidence. Peptide intensities were collected from extracted ion profiles from the MS1 profiles from the confident proteins identified and tabulated as abundances for the 3 technical replicates.

Center #3: In solution digestion for the sample was performed using an S-Trap micro spin column (ProtiFi, Huntington, NY, USA) following S-Trap protocol as described previously[2, 3] with slight modification. The sample solution was centrifuged at 12,000xg for 5 min to pellet the beads. The PBS storage solution was pipetted off and saved. The bead pellets were then incubated at room temp for 30 min with 25 µL buffer containing 50 mM triethylammonium bicarbonate (TEAB) pH 8.5, 6 M Urea, 2 M Thiourea, 4% SDS, 10 mM Dithiothreitol (DTT). The sample was centrifuged again at 12,000xg for 5 min to pellet the beads. The supernatant was removed and incubated for an additional 30 min at 34 °C to complete the reduction step, then alkylated with 65 mM iodoacetamide

for 30 min in dark and then quenched with a final concentration of 35 mM DTT. After quenching, 12% phosphoric acid was added to the sample for a final concentration of 1.2%, followed by 1:7 dilution (v/v) with a solvent containing 90% methanol and 0.1 M TEAB pH 8.5. The resulting sample was then placed into a S-Trap micro unit and centrifuged at 3000xg for 30 s. Samples were then washed three times with 150 µl 90% methanol, 0.1 M TEAB pH 8.5. Digestion was performed by adding 25 µl trypsin (40 ng/µl) in 50 mM TEAB pH 8.5 to the top of the spin column. The spin column was incubated overnight (16 h) at 37 °C. Following incubation, the digested peptides were eluted off the S-Trap column sequentially with 40 µl each of 50 mM TEAB pH 8.5 followed by 0.2% formic acid and finally, 50% acetonitrile, 0.2% formic acid. Three eluates with eluted peptides were pooled together and evaporated to dryness by a Speedvac SC110 (Thermo Savant, Milford, MA).

The tryptic digest was reconstituted in 73 µL of 0.5% formic acid (FA) for nanoLC-ESI-MS/MS analysis and divided to three separate vials. The analysis was carried out using an Orbitrap Fusion Tribrid (Thermo-Fisher Scientific, San Jose, CA) mass spectrometer equipped with a nanospray Flex Ion Source, and coupled with a Dionex UltiMate 3000 RSLCnano system (Thermo, Sunnyvale, CA).[4] The peptide samples (20 µL) of each aliquot were injected onto a PepMap C-18 RP nano trapping column (5 µm, 100 µm i.d. x 20 mm) at 20 µL/min flow rate for rapid sample loading and then separated on a PepMap C-18 RP nano column (2 µm, 75 µm i.d. x 25 cm) at 35 °C. The tryptic peptides were eluted in a 90 min gradient of 5% to 35% ACN in 0.1% formic acid at 300 nL/min, followed by a 7 min ramping to 90% ACN-0.1% FA and an 8 min hold at 90% ACN-0.1% FA. The column was re-equilibrated with 0.1% FA for 25 min prior to the next run. The Orbitrap Fusion was operated in positive ion mode with spray voltage set at 1.1 kV and source temperature at 275 °C. External calibration for FT, IT and quadrupole mass analyzers were performed. In data-dependent acquisition (DDA) analysis, the instrument was operated using FT mass analyzer in MS scan to select precursor ions followed by 3 S “Top Speed” data-dependent CID ion trap MS/MS scans at 1.6 m/z quadrupole isolation for precursor peptides with multiple charged ions above a threshold ion count of 10,000 and normalized collision energy of 30%. MS survey scanned at a resolving power of 120,000 (fwhm at m/z 200), for the mass range of m/z 375-1575. Dynamic exclusion parameters were set at 50 s of exclusion duration with ±10 ppm exclusion mass width. All data were acquired under Xcalibur 4.4 operation software (Thermo-Fisher Scientific).

The DDA raw files with MS and MS/MS were subjected to database searches using Proteome Discoverer (PD) 2.4 software (Thermo Fisher Scientific, Bremen, Germany) with the Sequest HT algorithm. The PD 2.4 processing workflow containing an additional node of Minora Feature Detector for precursor ion-based quantification was used for protein identification and relative quantitation of identified peptides and their modified forms. The database search was conducted against a *Homo Sapiens* NCBI database. The peptide precursor tolerance was set to 10 ppm and fragment ion tolerance was set to 0.6 Da. Variable modifications of methionine oxidation,

deamidation of asparagine/glutamine, acetylation, methionine loss and methionine loss plus acetylation on protein N-terminus and fixed modification of cysteine carbamidomethylation were set for the database search. Only high confidence peptides defined by Sequest HT with a 1% FDR by Percolator were considered for confident peptide identification. Relative quantitation of identified proteins between the three replicates was determined by the Label Free Quantitation (LFQ) workflow in PD 2.4. The precursor abundance intensity for each peptide identified by MS/MS in each replicate was automatically determined and their unique plus razor peptides for each protein in each replicate were summed and used for calculating the protein abundance by PD 2.4 software.

Center #4: 10 µl (20 ng/µl) of modified sequencing-grade trypsin (Promega, Madison, WI) was spiked into 300 µl PBS and the samples were placed in 37 ºC overnight. Samples were acidified by spiking in 20 µl of 20% formic acid solution and then desalted by STAGE tip.[1] On the day of analysis, the samples were reconstituted in 10 µl of HPLC solvent A. A nano-scale reverse-phase HPLC capillary column was created by packing 2.6 µm C18 spherical silica beads into a fused silica capillary (100 µm inner diameter x ~30 cm length) with a flame-drawn tip.[5] After equilibrating the column each sample was loaded via a Famos auto sampler (LC Packings, San Francisco CA) onto the column. A gradient was formed, and peptides were eluted with increasing concentrations of solvent B (97.5% acetonitrile, 0.1% formic acid). As peptides eluted, they were subjected to electrospray ionization and then entered into an LTQ Orbitrap Velos Elite ion-trap mass spectrometer (Thermo Fisher Scientific, Waltham, MA). Peptides were detected, isolated, and fragmented to produce a tandem mass spectrum of specific fragment ions for each peptide. Peptide sequences (and hence protein identity) were determined by matching protein databases with the acquired fragmentation pattern by the software program, Sequest (Thermo Fisher Scientific, Waltham, MA).[6] All databases include a reversed version of all the sequences and the data were filtered to between a one and two percent peptide false discovery rate.

Center #5: For protein digestion, the coated particles were washed twice with 50 µl of 100 mM of ammonium bicarbonate solution and transferred to a new tube and washed again twice more with the same solution. After the last spin, the protein bound-coated particles were incubated with 10 ng of sequencing grade modified trypsin (Promega) in 50 µl, and after brief vortex, were incubated overnight at 37 °C. Then coated particles were centrifuged on a tabletop centrifuge, The coated particles were washed one more time with 50 µl of 100 mM ammonium bicarbonate and supernatants were collected and acidified with formic acid to a final concentration of 5%.

For HPLC online tandem mass spectrometry analysis, the digested peptide mixture was split in three aliquots and resuspended in 10 µL of 0.1 % formic acid. Each aliquot was loaded on a reversed phase C18 column (3um, 100A) peptide trap column (Aclaim PepMap 75 um x 2cm) and after a 10 min wash with 0.1% formic acid, the peptide trap was connected to a reversed phase C18 (3 µm, 100) column (Aclaim PepMap 50 um

x 15 cm) connected online to an orbitrap Fusion Lumos Tribrid mass spectrometer. The peptides were eluted with the following gradient of solvent B (80% acetonitrile, 0.1% formic acid) in solvent B+A (solvent A was 0.1% formic acid, 3% acetonitrile): from 2% to 25 % B in 70 min, 25% to 40% B in 15 min, 40% to 90% in 1 min, followed by a final wash with 90% B for 10 min. The eluting peptides were ionized by ESI at 2700 volts and the transfer tube temperature was set to 275 °C. MS data acquisition was done in data dependent mode, working at top speed on a 3 s cycle. MS were acquired in profile mode in the m/z range 375 to 1500 on the orbitrap analyzer at 120,000 resolution using 50 ms maximum scan time and 120 s exclusion time, mass tolerance was set to 10 ppm. Tandem Mass scans were obtained by HCD at 35% collision energy in centroid mode, with orbitrap detection at a resolution of 30,000 and a maximum ion time of 54 ms.

For data analysis, all MSMS scans are searched using Protein Discoverer v.2.4 running Sequest HT against a human protein database (downloaded from UniProt on 7/2/2019). Full trypsin specificity was defined with a maximum of three missed cleavages and a precursor mass tolerance of 20 ppm and a fragment tolerance of 0.02 Da. Met oxidation, deamidation of Asn and Gln, phosphorylation of S, T and Y and Acetyl protein N-terminal modification were defined as variable modifications, with a maximum of three equal modifications and to 4 dynamic modifications. For false discovery rate (FDR), a concatenated reversed protein database was used, and peptide and protein false discovery rate were set up at 5% and validation was based on the q-value obtained from Percolator, with a p significance set at 0.05.

Center #6: All reagents were from Fisher Scientific. To improve digestion efficiency and proteome yield, the nanoparticles were first pelleted by centrifugation at 16K x g for 20 min and the supernatant transferred to fresh tube. The NP pellet was resuspended in 20 µl of urea buffer (6 M urea, 2 M thiourea, 100 mM ammonium bicarbonate, pH 8.0). The supernatant was supplemented with 1/10th volume of urea buffer. Samples were then reduced and alkylated and digested with 1 µg of trypsin (Promega V5111; Lot# 0000475640) according to our SOP (available upon request). Following digestion, peptides were desalted and concentrated using C18 100 µL tips (Pierce Cat# 87784; Lot#wa319454), lyophilized, and resuspended in 10 µL of 5% ACN, 0.1% FA. Note: the resuspension volume for the supernatant yielded an approximate 30-fold concentration of the sample.

A 1 µl injection was made, in triplicates, directly onto a 20 cm long x 75 µm inner diameter pulled-needle analytical column packed with Waters BEH-C18, 1.7 µm reversed phase resin. Peptides were separated and eluted from the analytical column with a gradient of acetonitrile at 300 nl/min. The Bruker nanoElute system is connected to a Bruker timsTOF-PRO mass spectrometer via a Bruker CaptiveSpray source.

LC gradient conditions: Initial conditions were 3% B (A: 0.1% formic acid in water, B: 99.9% acetonitrile, 0.1% formic acid), followed by 15 min ramp to 17% B, 17-25% B over 25 min, 25-37% B over 15 min, 37-80% B over 10 min, hold at 80% B for 20

min, ramp back (1 min) and hold (4 min) at initial conditions. Total run time was 90 min. (Internal reference: “Notrap_90min_BEHC18_20cmx75um”).

MS data were collected in positive-ion data-dependent PASEF mode over a m/z range of 100 to 1700, last calibration date: 11/23/2021. PASEF and TIMS were set to “on”. One MS and ten PASEF frames were acquired per cycle of 1.1 s (~1 MS and 120 MS/MS). Target MS intensity for MS was set at 10,000 counts/sec with a minimum threshold of 1000 counts/s. A charge-state-based rolling collision energy table was used from 76-123% of 42.0 eV. An active exclusion/reconsider precursor method with release after 0.4 min was used. If the precursor (within mass width error of 0.015 m/z) was >4X signal intensity in subsequent scans, a second MSMS spectrum was collected. Isolation width was set to 2 m/z (<700m/z) or 3 (800-1500 m/z). (Internal reference: “StandardPASEF_Cal-2021”).

The data were copied to our PEAKS-XPro server and searched against UniProt-Human (20,379 entries; last update 03082021) using the following parameters: trypsin semi-specific as enzyme, 2 missed cleavages allowed; 20 ppm mass error on precursor, 0.1 Da mass error on CID MSMS fragments; carbamidomethyl-Cys fixed modification; oxidized-Met, deamidated-N/Q as variable modifications. Data were then filtered as follows: all identified peptides were filtered for p<0.1 peptide false discovery rate; show protein groups only (remove redundant DB entries), and ≥2 spectral counts.

Center #7: Samples were brought to a total volume of 100 µL 6 M urea, 100 mM tris pH 7.8. Reduction and alkylation of disulfide bonds was then carried out by the addition of 5 µl 200 mM dithiothreitol (DTT) for 60 min to reduce disulfide bonds. The resulting free cysteine residues were subjected to an alkylation reaction by the addition of 20 µl 200 mM iodoacetamide for 60 min to form carbamidomethyl cysteine. This solution was brought to a volume of 900 µl to reduce the urea concentration. 100 µl of 20 ng/µl of trypsin or chymotrypsinin was added for a final volume of 1000 µl. These were allowed to digest overnight at 37 °C with gentle shaking. The resulting peptides were washed, extracted and concentrated by solid phase extraction using Waters Sep-Pak Plus C18 cartridges organic solvent was removed and the volumes were reduced to 50 µL using a speed vac for subsequent analyses. Three 10 µl injections of the digested extract was analyzed by reversed phase high performance liquid chromatography (HPLC) using Waters NanoAcquity pumps and autosampler and a ThermoFisher Orbitrap Elite mass spectrometer using a nano flow configuration. A 20 mm x 180 µm column packed with 5 µm Symmetry C18 material (Waters) using a flow rate of 15 µl per min for two min was used to trap and wash peptides. These were then eluted onto the analytical column which was a self-packed with 3.6 µm Aeris C18 material (Phenomenex) in a fritted 20 cm x 75 µm fused silica tubing pulled to a 5 µm tip. The gradient was isocratic 1% A Buffer for 1 min 250 nl min-1 with increasing B buffer concentrations to 15% B at 42.5 min, 27% B at 62 min and 40% B at 73.5 min. The column was washed with high percent B and re-equilibrated between analytical runs for a total cycle time of approximately 97 min. Buffer A consisted of 1% formic acid in water and buffer B consisted of 1% formic acid in

acetonitrile.

The mass spectrometer was operated in a data-dependent acquisition mode where the 10 most abundant peptides detected in the Orbitrap using full scan mode with a resolution of 240,000 were subjected to daughter ion fragmentation in the linear ion trap. A running list of parent ions was tabulated to an exclusion list to increase the number of peptides analyzed throughout the chromatographic run. Peptides were identified from the MS data using PEAKS Studio 10plus (Bioinformatic Solutions, Inc) algorithms. Refseq entries for *Homo sapiens* were downloaded from NCBI and concatenated to a database of common contaminants (keratin, trypsin, etc). An additional database consisting of 60 sequences was concatenated to the same contaminant database. A peptide threshold of 99.5% was used as cutoffs for identification of peptides and proteins.

Center #8: Nanoparticles were resuspended in 200 µl of 50 mM TEAB prior to reduction in 10 mM DTT followed by alkylation using 30 mM acrylamide to cap cysteine residues. Digestion was performed using Trypsin/LysC (Promega) in the presence of 0.02% ProteaseMax (Promega) overnight. Following digestion and quenching, peptides were desalted, dried, and reconstituted in 2% aqueous acetonitrile prior to analysis.

Mass spectrometry experiments were performed on a Q Exactive HF-X Hybrid Quadrupole - Orbitrap mass spectrometer (Thermo Scientific, San Jose, CA) connected to a Nanoacquity UPLC system (Waters Corporation, Milford, MA). The UPLC system was set to a flow rate of 300 nl/min, where mobile phase A was 0.2% formic acid in water and mobile phase B was 0.2% formic acid in acetonitrile. The analytical column was prepared in-house with an I.D. of 100 microns pulled to a nanospray emitter using a P2000 laser puller (Sutter Instrument, Novato, CA). The column was packed with NanoLCMS solutions 1.8 µm C18 stationary phase to a length of approximately 25 cm. Peptides were directly injected into the column with a gradient of 2-45% mobile phase B, followed by a high-B wash over a total of 80 min. The mass spectrometer was operated in a data dependent fashion using HCD fragmentation for MS/MS spectra generation.

RAW data were analyzed using Byonic v4.2.4 (Protein Metrics, Cupertino, CA) to identify peptides and infer proteins. A concatenated FASTA file containing the UniProt *Homo sapiens* sequences and other likely contaminants and impurities was used. Proteolysis with Trypsin/LysC was assumed to be semi-specific allowing for N-ragged cleavage with up to two missed cleavage sites. Both precursor and fragment mass accuracies were held within 12 ppm. Cysteine modified with propionamide was set as a fixed modification in the search. Variable modifications included oxidation on methionine, histidine and tryptophan, dioxidation on methionine and tryptophan, deamidation on asparagine and glutamine, and acetylation on protein N-terminus. Proteins were held to a false discovery rate of 1% using standard reverse-decoy technique.[7]

Center #9: 250 µl of sample suspension was mixed with 25 µl of 10% SDS (0.91% final concentration), incubated at 50 °C for 30 min to extract proteins, cooled down

to room temperature (RT), centrifuged at 16,000xg for 20 min at RT, and 240 µl supernatant was collected for further processing. Assumption: collected 240 µl supernatant (extract) may contain up to (less than) 6 µg of protein. Collected 240 µl of protein extract was mixed with 13 µl of 1 M triethylammonium bicarbonate (TEAB), ~50 mM final concentration). The extracted proteins were reduced with 5 mM DTT at 50 °C for 45 min, alkylated with 20 mM iodoacetamide (IAM) at RT for 20 min in the dark, and incubated with 5 volumes (1350 µl) of precooled acetone at -20°C overnight for precipitation; the mixture was centrifuged at 16,000xg for 10 min at 4 °C, and the protein pellet was collected. The protein pellet was dissolved in 200 µl of 10 mM triethylammonium bicarbonate (TEAB), 5 volumes (1000ul) of precooled acetone was added and overnight precipitation at -20 °C was repeated. The collected protein pellet was air dried at RT for 4 min, dissolved in 75 µl 100 mM TEAB and the proteins were digested overnight at 37 °C with 0.4 µg of Pierce Trypsin/Lys-C Protease mixture (cat. # A41007, Thermo Fisher). The digested material was vacuum dried and desalted using Pierce C-18 spin tips (cat. # 84850) according to the manufacturer's protocol. The desalted peptide sample was dried in SpeedVac for 60 min and dissolved in 50 µl of loading buffer (3% acetonitrile wit 0.1% TFA) for LC/MS/MS analysis.

5 µl aliquots (less than 0.6 µg) of the digested/desalted material were analyzed in triplicate for peptide/protein identification and label free quantitation using HPLC in line with tandem mass spectrometry. Used instrumentation and parameters are specified bellow:

HPLC: Ultimate 3000RSLCnano, Thermo Fisher
Trap column: Acclaim PepMap 100, 75µm x 20mm, C18, 3µm, 100Å, Thermo Fisher
Column: Acclaim PepMap RSLC, 75µm x 500mm (ID x Length), C-18, 2µm, 100Å, Thermo Fisher
Injection volume/mode: 5µl/µl PickUp
Loading Buffer: 3% acetonitrile with 0.1% TFA
Loading flow rate and duration: 5µl/min for 5min
Solvent A: 0.1% formic acid in water, LC/MS grade, Thermo Fisher
Solvent B: 0.1% formic acid in acetonitrile, LC/MS grade, Thermo Fisher
LC flow rate: 300 nl/min
Column temperature: 40 °C
Gradient: 0min-3%B, 4min-3%B, 5min-5%B, 55min-25%B, 60min-30%B, 63min-90%B, 73min-90%B, 76min-3%B, 100min-3%B
MS: Orbitrap Fusion Lumos, Thermo Fisher
Data dependent analysis (DDA): 3sec cycles
MS scan (full): Analyzer - Orbitrap, resolution-120,000 (FWHM, at m/z=200)
Scan Filters: MIPS mode - Peptide
Intensity threshold ≥ 10,000
Charge state - 2-6

Dynamic exclusion – 30 sec
MS2 scan (full): Quadrupole isolation window - 0.7 m/z,
Activation - HCD (30%)
Analyzer - Orbitrap, Resolution 30,000 (FWHM, at m/z=200)
Post-Acquisition Analysis
Proteome Discoverer 2.4, Thermo Fisher
`Peptide/Protein Identification
Search engine: Sequest HT
Database: SwissProt, TaxID 9606 (Homo sapiens), v.2017-05-10, 42153 entries
Enzyme: Trypsin (full)
Dynamic modification: Oxidation of Met, acetylation of the protein N-terminus
Static modification: Carbamidomethylation of Cys
Precursor and fragment ion mass tolerance: 10 ppm and 0.02 Da, respectively
Validation and filtering at PSM level (q value): Percolator, FDR ≤0.01
Identification of protein or protein group: At least one validated peptide sequence unique to a protein or a protein group
Protein groups: Strict parsimony principle applied
Validation at protein level (Experimental q value): strict - FDR≤0.01, relaxed - FDR≤0.05
Feature Detection
Min Trace Length: 5
Min # Isotopes: 2
Max ΔRT of Isotope Pattern Multiplets: 0.2min
Chromatographic Alignment
Max RT shift: 5 min
Mass tolerance: 10 ppm
Feature Linking/Mapping
RT tolerance: 0 (automatic)
Mass tolerance: 0 (automatic)
Min S/N threshold: 5
Peptide/Protein Quantification:
Quantification: LFQ - Label-free Quantification (Precursor Ion Area Detection)
Peptides to use: Unique + Razor
Peptide uniqueness: Protein Group
Peptide Abundance: MS Peak Area
Normalization mode: Total Peptide Amount
Peptide abundance: Summed abundances of assigned PSMs
Protein abundance: Summed abundances of assigned peptides

Peptide Group abundance: Mean of replicate abundances

Protein Group abundance: Mean of replicate abundances

Center #10: Protein samples were diluted in TNE (50 mM Tris pH 8.0, 100 mM NaCl, 1 mM EDTA) buffer. RapiGest SF reagent (Waters) was added to the mix to a final concentration of 0.1% and samples were boiled for 5 min. TCEP (Tris (2-carboxyethyl) phosphine) was added to 1 mM (final concentration) and the samples were incubated at 37 °C for 30 min. Subsequently, the samples were carboxymethylated with 0.5 mg/ml of iodoacetamide for 30 min at 37 °C followed by neutralization with 2 mM TCEP (final concentration). Proteins samples prepared as above were digested with trypsin (trypsin:protein ratio - 1:50) overnight at 37 °C. RapiGest was degraded and removed by treating the samples with 250 mM HCl at 37 °C for 1 h followed by centrifugation at 14,000xg for 30 min at 4 °C. The soluble fraction was then added to a new tube and the peptides were extracted and desalted using C18 desalting columns (Thermo Scientific, PI-87782). Peptides were quantified using BCA assay and a total of 1 µg of peptides was injected for LC-MS analysis.[8]

Trypsin-digested peptides were analyzed by ultra-high pressure liquid chromatography (UPLC) coupled with tandem mass spectroscopy (LC-MS/MS) using nano-spray ionization. The nanospray ionization experiments were performed using an Orbitrap fusion Lumos hybrid mass spectrometer (Thermo) interfaced with nano-scale reversed-phase UPLC (Thermo Dionex UltiMate™ 3000 RSLC nano System) using a 25 cm, 75-micron ID glass capillary packed with 1.7-µm C18 (130) BEHTM beads (Waters corporation). Peptides were eluted from the C18 column into the mass spectrometer using a linear gradient (5–80%) of ACN (Acetonitrile) at a flow rate of 375 µl/min for 1.5 h. The buffers used to create the ACN gradient were: Buffer A (98% H2O, 2% ACN, 0.1% formic acid) and Buffer B (100% ACN, 0.1% formic acid). Mass spectrometer parameters are as follows; an MS1 survey scan using the orbitrap detector (mass range (m/z): 400-1500 (using quadrupole isolation), 120,000 resolution setting, spray voltage of 2200 V, Ion transfer tube temperature of 275 C, AGC target of 400,000, and maximum injection time of 50 ms) was followed by data dependent scans (top speed for most intense ions, with charge state set to only include +2-5 ions, and 5 s exclusion time, while selecting ions with minimal intensities of 50,000 at in which the collision event was carried out in the high energy collision cell (HCD Collision Energy of 30%), and the fragment masses where analyzed in the ion trap mass analyzer (With ion trap scan rate of turbo, first mass m/z was 100, AGC Target 5000 and maximum injection time of 35ms). Protein identification was carried out using Peaks Studio 8.5 (Bioinformatics solutions Inc).[9]

Center #11: Sample was centrifuged at 21,000 x g for 10 min and the PBS removed. 50 µl of 1X NuPAGE™LDS reducing (5 mM DTT) sample buffer was added and the beads resuspended before heating at 95 °C for 10 min with gentle shaking. They were then centrifuged again at 21,000 x g for 10 min and the sample separated from the beads. 45 of the 50 µl was then loaded onto a 12% BOLT™ SDS-PAGE gel (Bolt™ 12%, Bis-Tris, 1.0 mm, Mini Protein Gel, 10-well, ThermoFisher Scientific) and the sample was run into the top 1 cm of the gel. The gel containing protein was excised, reduced with DTT

and alkylated with iodoacetamide. Gel samples were then washed, destained and the proteins digested with 250 ng trypsin (Promega sequencing grade cat# V5111) overnight at 37 °C. Peptides were extracted from the gel pieces, dried down, and re-dissolved in 30 µl 5% acetonitrile, 0.5% trifluoroacetic acid. 3 x 5 µl injections were run by nanoLC-MS/MS using a 2 h method on a CSH 0.075 mm x 250 mm C18 column (Waters Corp, Milford, MA) feeding into an Orbitrap Eclipse mass spectrometer (Thermofisher) running in OT-OT mode.

The quantitation of the proteins was done using Proteome Discoverer (Thermofisher; version 2.4). All MS/MS samples were searched using Mascot (Matrix Science, London, UK; version 2.7.0). Mascot was set up to search the cRAP_20150130.fasta (125 entries); UniProt-human_20210508 database (77027 entries) assuming the digestion enzyme trypsin. Mascot was searched with a fragment ion mass tolerance of 0.02 Da and a parent ion tolerance of 10.0 PPM. Deamidation of asparagine and glutamine, as well as oxidation of methionine were specified in Mascot as variable modifications. Carbamidomethylation of cysteine was specified as a fixed modification. Peptides were validated by Percolator with a 0.01 posterior error probability (PEP) threshold. The data were searched using a decoy database to set the false discovery rate to 1% (high confidence). The peptides were quantified using the precursor abundance based on intensity. The peak abundance was normalized using total peptide amount. The peptide group abundances are summed for each sample and the maximum sum for all files is determined. The normalization factor used is the factor of the sum of the sample and the maximum sum in all files. Scaled abundances are also included. The abundances are adjusted so that the average of the abundances is equal to 100 for each sample.

Center #12: A 300 µl sample was divided in equal aliquots of 100 µl each and each buffered by adding triethylammonium bicarbonate (TEAB) buffer (Honeywell Fluka cat# 60-044-974) for a final concentration of 20 mM. Samples were reduced by adding 5 mM DL-Dithiothretol (DTT, Sigma cat# D5545) and incubated 30 min at 37 °C. Alkylation was performed by adding 15 mM Iodoacetamide (IAA, Sigma cat# I1149) and incubating samples at room temperature for 30 min in the dark. The alkylation reaction was stopped by adding 5 mM DTT to the samples. Tryptic digest was initiated by adding 0.35 µg of trypsin (Promega, V5113) per tube then incubating 1 h at 47 °C followed by 3 hours at 37 °C. Digestion was assessed by running a small aliquot of each sample on LC-MS/MS. Final analysis was performed on the same samples using a Thermo scientific Easy-nLC 1000 chromatography system with a C18 Acclaim PepMap 100 trap column, 75 µm x 2 cm (Thermo scientific) and Acclaim PepMap RSLC, 75 µm x 25 cm column (Thermo scientific). LC-MS/MS was performed using Data Dependent Analysis on an Orbitrap Fusion MS system. MS1 spectra were acquired at 120,000 resolution and MS2 in the ion trap. Data were analyzed using Proteome Discoverer 2.4 using Sequest NT and Percolator algorithms searching a human database downloaded on 3/30/2021 (UniProt UP000005640) with 20,310 protein entries. Parameters in the search were set to accept up to 2 missed cleavages by trypsin digestion. Carbamidomethylation of

Cysteine was a fixed modification and dynamic modifications were deamination of asparagine and glutamine as well as oxidation of methionine. An additional dynamic N-terminus acetyl modification was also applied. False Discovery Rate (FDR) was set at 0.01 for high confidence matches.

Center #13: Sample in 1X PBS was digested with 0.5 microgram of trypsin (Thermo Pierce, Proteomics Grade) for 30 min at 55 °C (60 watts) using a CEM Discover Microwave Digestor (Matthews, NC). The digested material was lyophilized, desalted using StageTips,[10] lyophilized and dissolved in 5% acetonitrile containing 0.1% formic acid before injection to LC/MS.

For LC/MS, 0.5 microgram of digested sample was used. The system used was a Thermo UltiMate 3000 UHPLC system coupled online to a high resolution Thermo Orbitrap Fusion Tribrid mass spectrometer. Peptides were separated by reversed-phase chromatography using a 50 cm μPAC C18 nano-LC column (PharmaFluids, Ghent, Belgium) with mobile phases of 0.1% formic acid (A) and 0.1% formic acid in acetonitrile (B); a linear gradient from 4% B to 35% B over the course of 90 min was employed for peptide separation, followed by additional steps for column washing and regeneration. The mass spectrometer was operated in a data dependent manner in which precursor scans from 300 to 2000 m/z (120,000 resolution) were followed by collision induced dissociation of the most abundant precursors over a maximum cycle time of 3 s (35% NCE, 1.6 m/z isolation window, 60 s dynamic exclusion window).[10]

The raw LC-MS/MS data was analyzed against the Swissprot database (2021-02) for *Homo sapiens* using Mascot 2.8 (London, UK). Tryptic digestion was specified with a maximum of 2 missed cleavages, while peptide and fragment mass tolerances were set to 10 ppm and 0.6 Da, respectively. Variable modifications included oxidation of methionine was added to the search.

Center #14: Total protein was determined on a Qubit and the appropriate volume of each sample was taken to equal 8 μg total protein for digestion. The samples were digested with sequencing grade trypsin/lys C rapid digestion kit from Promega (Madison WI) using manufacture recommended protocol. Three times the sample volume of rapid digestion buffer (provided with the kit) was added to the samples. The sample was incubated at 56 °C with 1 μL of dithiothreitol (DTT) solution (0.1 M in 100 mM ammonium bicarbonate) for 30 min prior to the addition of 0.54 μL of 55 mM iodoacetamide in 100 mM ammonium bicarbonate. Iodoacetamide was incubated at room temperature in dark for 30 min. The trypsin/lys C was prepared fresh as 1 μg/μL in the rapid digestion buffer. 1 μL of enzyme was added and the samples were incubated at 70 °C for 1 h. The digestion was stopped with addition of 0.5% TFA. The MS analysis was immediately performed to ensure high quality tryptic peptides with minimal non-specific cleavage.

Nano-liquid chromatography tandem mass spectrometry (Nano-LC/MS/MS) was performed on a Thermo Scientific Q Exactive HF Orbitrap mass spectrometer equipped with an EASY Spray nanospray source (Thermo Scientific) operated in positive ion

mode. The LC system was an UltiMate™ 3000 RSLCnano system from Thermo Scientific. The mobile phase A was water containing 0.1% formic acid and the mobile phase B was acetonitrile with 0.1 % formic acid. The mobile phase A for the loading pump was water containing 0.1 % trifluoracetic acid. 5 ml of sample is injected on to a PharmaFluidics mPAC™ C18 trapping column (C18, 5 µm pillar diameter, 10 mm length, 2.5 µm inter-pillar distance). at 10 µl/ml flow rate. This was held for 3 min and washed with 1 % B to desalt and concentrate the peptides. The injector port was switched to inject and the peptides were eluted off of the trap onto the column. PharmaFluidics 50 cm mPAC™ was used for chromatographic separations (C18, 5 µm pillar diameter, 50 cm length, 2.5 µm inter-pillar distance). The column temperature was maintained 40 °C. A flowrate of 750 nl/min was used for the first 15 min and then the flow was reduced to 300 nl/min. Peptides were eluted directly off the column into the Q Exactive HF Orbitrap system using a gradient of 1% B to 20% B over 100 min and then to 45% B in 20 min for a total run time of 150 min:

Time (min)	% B	Flow Rate (nL/min)
0	1	750
3	1	750
15	5	750
15.1	5	300
100	20	300
123	45	300
130	95	300
135	95	300
135.1	1	300
150	1	300

The total run time was 150 min. The MS/MS was acquired according to standard conditions established in the lab. The EASY Spray source operated with a spray voltage of 1.5 KV and a capillary temperature of 200 °C. The scan sequence of the mass spectrometer was based on the original TopTen™ method; the analysis was programmed for a full scan recorded between 375 – 1575 Da at 60,000 resolution, and a MS/MS scan at resolution 15,000 to generate product ion spectra to determine amino acid sequence in consecutive instrument scans of the fifteen most abundant peaks in the spectrum. The AGC Target ion number was set at 3e6 ions for full scan and 2e5 ions for MS^2 mode. Maximum ion injection time was set at 50 ms for full scan and 55 ms for MS^2 mode. Micro scan number was set at 1 for both full scan and MS^2 scan. The HCD fragmentation energy (N)CE/stepped NCE was set to 28 and isolation window to 4 *m/z*. Singly charged ions were excluded form MS^2. Dynamic exclusion was enabled with a repeat count of 1 within 15 s and to exclude isotopes. A Siloxane background peak at 445.12003 was used as the internal lock mass. HeLa protein digest standard is used to evaluate the integrity and the performance of the columns and mass spectrometer. If the number of protein ID's from the HeLa standard falls below 2700, the instrument is cleaned and new columns are

installed. All MS/MS samples were analyzed using Sequest (Thermo Fisher Scientific, San Jose, CA, USA; version IseNode in Proteome Discoverer 2.4.0.305). Sequest was set up to search against a *Homo sapiens* database (NcbiAV TaxID=9606) (v2017-10-30) assuming the digestion enzyme trypsin. Sequest was searched with a fragment ion mass tolerance of 0.020 Da and a parent ion tolerance of 10.0 ppm. Carbamidomethylation of cysteine was specified in Sequest as a fixed modification. Loss of methionine, Loss of methionine+Acetyl of methionine, oxidation of methionine and acetyl of the n-terminus were specified in Sequest as variable modifications. CRITERIA FOR PROTEIN IDENTIFICATION-- Scaffold (version Scaffold_4.11.1, Proteome Software Inc., Portland, OR) was used to validate MS/MS based peptide and protein identifications. Peptide identifications were accepted if they could be established at greater than 95.0% probability by the Percolator posterior error probability calculation.[11] Protein identifications were accepted if they could be established at greater than 99.0% probability and contained at least 1 identified peptide. Protein probabilities were assigned by the Protein Prophet algorithm.[12] Proteins that contained similar peptides and could not be differentiated based on MS/MS analysis alone were grouped to satisfy the principles of parsimony. Proteins sharing significant peptide evidence were grouped into clusters. Protein quantitation was performed on the paired data using Fischer's Exact test with a Benjamini-Hochberg correction applied to calculate *P* values. Fold change was determined using weighted spectra.

Center #15: Protein sample was reduced, alkylated with iodoacetamide, and digested with a trypsin/Lys-C protease mixture using Thermo Scientific EasyPep Mini MS Sample prep kit (Cat #A40006) and following the provided protocol.

Trap Method

No	Time	Flow [µl/min]	%B	%C	Curve
1	0.000	Equilibration			
2	0.000	10.000	0.0	100.0	5
3	*New Row*				
4	0.000	Run			
5	0.000	10.000	0.0	100.0	5
6	5.000	10.000	0.0	100.0	5
7	138.000	2.000	0.0	100.0	5
8	*New Row*				
9	138.000	Stop Run			

Separation Gradient

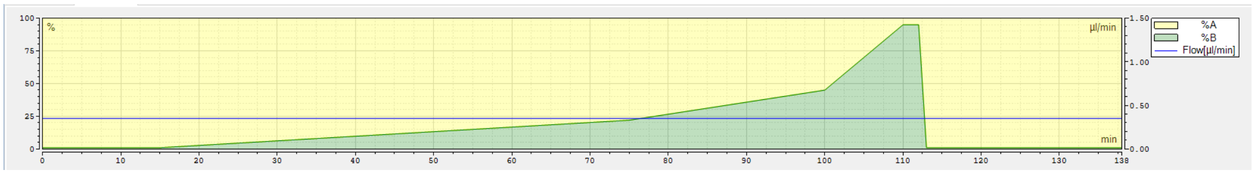

No	Time	Flow [µl/min]	%B	Curve
1	0.000	Equilibration		
2	0.000	0.350	1.0	5
3	*New Row*			
4	0.000	Run		
5	0.000	0.350	1.0	5
6	15.000	0.350	1.0	5
7	75.000	0.350	22.0	5
8	100.000	0.350	45.0	5
9	110.000	0.350	95.0	5
10	112.000	0.350	95.0	5
11	113.000	0.350	1.0	5
12	138.000	0.350	1.0	5
13	*New Row*			
14	138.000	Stop Run		

Sample were cleaned up for analysis using the column provided with the kit. Samples were analyzed using an UltiMate 3000 RSLCnano system (Thermo Scientific, San Jose, CA). The peptides were trapped prior to separation on a 300 µm i.d. x 5 mm C18 PepMap 100 trap (Thermo Scientific, San Jose, CA) for 5 min at 10 µl/min. Separation was performed on a 50 cm µPAC C18 nano-LC column (PharmaFluidics, Ghent, Belgium) with a 20 µm fused silica emitter with a Nanospray Easy-Spray ion source (Thermo Scientific, San Jose, CA). Separation was performed at 350 nl/min using a gradient from 1% - 45% in the above graph (Solvent A 0.1% Formic Acid, Solvent B Acetonitrile, 0.1% Formic Acid).

Mass spectral analysis was performed using an Orbitrap Eclipse mass spectrometer (Thermo Scientific, San Jose, CA). The MS1 precursor selection range is from 375-1500 m/z at a resolution of 120K with a normalized automatic gain control (AGC) target of 250% and an automatic maximum injection time. Quadrupole isolation of 0.7 Th for MS2 isolation and CID fragmentation in the linear ion trap with a collision energy of 35% and a 10 ms activation time. The MS2 AGC was in standard mode with a 35 ms maximum injection time. The instrument was operated in a data-dependent mode with a 3 s cycle time and the most intense precursor priority, and the dynamic exclusion set to an exclusion duration of 60 s with a 10 ppm tolerance.

All MS/MS samples were analyzed using Sequest (Thermo Fisher Scientific, San Jose, CA, USA; version IseNode in Proteome Discoverer 2.2.0.388). Sequest was set up to search UniProt-homo_sapiens_20190201.fasta (147857 entries) assuming the digestion enzyme trypsin. Sequest was searched with a fragment ion mass tolerance of 0.60 Da and a parent ion tolerance of 10.0 PPM. Carbamidomethyl of cysteine was specified in Sequest as a fixed modification. Oxidation of methionine, acetyl of the n-terminus and phosphorylation of serine were specified in Sequest as variable modifications.

Scaffold (version Scaffold_5.0.1, Proteome Software Inc., Portland, OR) was used to validate MS/MS based peptide and protein identifications. Peptide identifications were accepted if they could be established at greater than 95.0% probability by the Peptide Prophet algorithm with Scaffold delta-mass correction.[13] Protein identifications were accepted if they could be established at greater than 95.0% probability and contained at least 2 identified peptides. Protein probabilities were assigned by the Protein Prophet algorithm.[14] Proteins that contained similar peptides and could not be differentiated based on MS/MS analysis alone were grouped to satisfy the principles of parsimony.

Center #16: Protein bound NPs, in PBS, were supplemented with acetonitrile to 10% and reduced by addition of 10 mM Pierce BondBreaker TCEP. The mixture was heated at 95 °C for 10 min with agitation (700rpm) using an Eppendorf ThermoMixer R. Samples were cooled to room temperature and alkylated by addition of chloroacetamide to 15 mM with incubation at room temperature in the dark for 60 min. Alkylation was quenched by addition of DTT to 5 mM. Trypsin, in 50 mM ammonium bicarbonate, was added to 30 ng and the samples incubated overnight at 37 °C. The solution was acidified to 1% with trifluoroacetic acid and desalted by C18 solid phase extraction using StageTips.[15] Purified peptides eluates were dried by vacuum centrifugation and frozen at -20 °C.

Samples were re-suspended in 2% acetonitrile/0.1% trifluoroacetic acid/97.9% water to 12 µl. An injection of 10 µl was automatically made using a Thermo (www.thermo.com) EASYnLC 1000 onto a Thermo Acclaim PepMap RSLC 0.1 mm x 20 mm C18 trapping column and washed for ~5 min with buffer A. Bound peptides were then eluted over 95 min onto a Thermo Acclaim PepMap RSLC 0.075 mm x 250 mm resolving column with a gradient of 5% B to 8% B at 2 min, 8% B to 28% B at 72 min, 28% B to 38% B at 84 min, ramping to 90% B at 85 min and held at 90% B for the duration of the run (Buffer A = 99.9% Water/0.1% Formic Acid, Buffer B = 80% Acetonitrile/0.1% Formic Acid/19.9% Water) at a constant flow rate of 300 nl/min. Column temperature was maintained at a constant temperature of 50 °C using and integrated column oven (PRSO-V1, Sonation GmbH, Biberach, Germany). Eluted peptides were sprayed into a Thermo Scientific Q-Exactive mass spectrometer (www.thermo.com) using a FlexSpray spray ion source. Survey scans were taken in the Orbitrap (35000 resolution, determined at m/z 200) and the top 15 ions in each survey scan are then subjected to automatic higher energy collision induced dissociation (HCD) with fragment spectra acquired at 17,500 resolution.

The resulting MS/MS spectra are converted to peak lists Mascot Distiller, v2.7.0 (www.matrixscience.com) and searched against all human protein entries available from UniProt (www.uniprot.org) appended with common laboratory contaminants (downloaded from www.thegpm.org, cRAP project) The Mascot output was then analyzed using Scaffold, v5.1 (www.proteomesoftware.com) to probabilistically validate protein identifications. Assignments validated using the Scaffold 1% FDR

confidence filter are considered true. Mascot parameters for all databases were as follows:

- Allow up to 2 missed tryptic sites
- Fixed modification of Carbamidomethylation on cysteine,
- Variable modification of Oxidation of Methionine,
- Peptide tolerance of +/- 10ppm
- MS/MS tolerance of 0.02 Da
- FDR calculated using randomized database search

Center #17: A 100 µL aliquot of the sample was mixed with an equal volume of 2x Laemmli sample buffer (Biorad) containing 2-mercaptoethanol and boiled for 5 min before loading onto a 12% Mini-PROTEAN® TGX™ Precast Gel to 1 cm and stained using Coomassie Brilliant Blue R-250. After destaining, the 1 cm bands were excised, pooled, and digested with proteomics-grade trypsin (Sigma-Aldrich) using the manufacturer's recommended protocol. Extracted tryptic peptides were evaporated to dryness using a Speedvac and reconstituted in 15 µL of 0.1% aqueous formic acid for LC-MS/MS analysis.

Three replicates of the trypsin-digested proteins (2 µL injections) were analyzed on a nano- reversed-phase liquid chromatography (LC) electrospray ionization tandem mass spectrometry (MS) system that consisted of an UltiMate 3000 Nano LC System and an LTQ-Orbitrap Elite mass spectrometer (Thermo Fisher, San Jose, CA). Chromatography was performed using a home-made 25 cm × 75 µm ID column packed with XBridgeTM BEH C18 beads (2.5 µm, 130 Å). Solvent A (0.1% aqueous formic acid) and B (0.1% formic acid in acetonitrile) were used to establish the 85 min gradient, comprised of 60 min of 5-45% B, then 15 min of 45-90% B, and finally maintained at 90% B for 10 min, with the flow rate at 200 nL/min. The LTQ-Orbitrap Elite mass spectrometer was operated in positive ionization mode with a 2.6 kV applied spray voltage. The temperature of the ion transfer capillary was 300 °C. One microscan was set for each MS and MS/MS scan. A full scan MS acquired in the range $300 \leq m/z \leq 2000$ was followed by 10 data dependent MS/MS events on the 10 most intense ions. The mass resolution was set at 60000 for full MS. The dynamic exclusion function was set as follows: repeat count, 1; repeat duration, 30 s; exclusion duration, 30s. HCD was performed at a normalized collision energy of 35% and the activation time was set at 0.1 ms.

LC-MS/MS data files were converted to mascot generic file format using ProteoWizard msConvert. Probability-based protein database searching of MS/MS spectra against the NCBI human protein database was performed using MASCOT (Matrix Science, London, UK) with the following search criteria: precursor ion mass tolerance, 25 ppm; product ion mass tolerance, 50 mmu; up to 5 trypsin missed cleavages, oxidation of methionine residues as a variable modification.[16]

Supplementary References

1. Rappsilber J, Ishihama Y, Mann M. Stop And Go Extraction tips for matrix-assisted laser desorption/ionization, nanoelectrospray, and LC/MS sample pretreatment in proteomics. *Analytical Chemistry* **75**, 663-670 (2003).

2. Yang Y, Anderson E, Zhang S. Evaluation of six sample preparation procedures for qualitative and quantitative proteomics analysis of milk fat globule membrane. *Electrophoresis* **39**, 2332-2339 (2018).

3. Zougman A, Selby PJ, Banks RE. Suspension trapping (STrap) sample preparation method for bottom-up proteomics analysis. *Proteomics* **14**, 1006-1000 (2014).

4. Harman RM, He MK, Zhang S, Van De Walle GR. Plasminogen activator inhibitor-1 and tenascin-C secreted by equine mesenchymal stromal cells stimulate dermal fibroblast migration in vitro and contribute to wound healing in vivo. *Cytotherapy* **20**, 1061-1076 (2018).

5. Peng J, Gygi SP. Proteomics: The move to mixtures. *Journal of Mass Spectrometry* **36**, 1083-1091 (2001).

6. Eng JK, McCormack AL, Yates JR. An approach to correlate tandem mass spectral data of peptides with amino acid sequences in a protein database. *Journal of the American Society for Mass Spectrometry* **5**, 976-989 (1994).

7. Elias JE, Gygi SP. Target-decoy search strategy for increased confidence in large-scale protein identifications by mass spectrometry. *Nature Methods* **4**, 207-214 (2007).

8. Guttman M, Betts Gn Fau - Barnes H, Barnes H Fau - Ghassemian M, Ghassemian M Fau - van der Geer P, van der Geer P Fau - Komives EA, Komives EA. Interactions of the NPXY microdomains of the low density lipoprotein receptor-related protein 1. *Proteomics* **22,** 5016-5028 (2009).

9. McCormack AL*, et al.* Direct analysis and identification of proteins in mixtures by LC/MS/MS and database searching at the low-femtomole level. *Analytical Chemistry* **69,** 767-776 (1997).

10. Käll L, Storey JD, Noble WS. Non-parametric estimation of posterior error probabilities associated with peptides identified by tandem mass spectrometry. *Bioinformatics* **24**, i42-i48 (2008).

11. Hsu JL, Huang Sy Fau - Chow N-H, Chow Nh Fau - Chen S-H, Chen SH. Stable-isotope dimethyl labeling for quantitative proteomics, *Analytical Chemistry* **75,** 6843-52 (2003).

12. Keller A, Nesvizhskii Ai Fau - Kolker E, Kolker E Fau - Aebersold R, Aebersold R. Empirical statistical model to estimate the accuracy of peptide identifications made by MS/MS and database search, *Analytical Chemistry* **74,** 5383-92 (2002).

13. Nesvizhskii AI, Keller A, Kolker E, Aebersold R. A statistical model for identifying proteins by tandem mass spectrometry. *Analytical Chemistry* **75**, 4646-4658 (2003).

14. Rappsilber J, Mann M, Ishihama Y. Protocol for micro-purification, enrichment, pre-fractionation and storage of peptides for proteomics using StageTips. *Nature Protocols* **2**, 1896-1906 (2007).

15. Chambers MC*, et al.* A cross-platform toolkit for mass spectrometry and proteomics. *Nature Biotechnology* **30**, 918-920 (2012).
