# Supplementary material for: Measurements of heterogeneity in proteomics analysis of the nanoparticle protein corona across core facilities
Source: Nat Commun. 2022 Nov 3;13:6610. doi: 10.1038/s41467-022-34438-8 (PMC9633814; doi:10.1038/s41467-022-34438-8)
Supplement: Supplementary file 3 — Description of Additional Supplementary Files [file 41467_2022_34438_MOESM3_ESM.pdf]

Description of Additional Supplementary Files

File Name: Supplementary Data 1:

Description: The compiled data for all proteomics analyses from 12 cores providing semiquantitative results.

File Name: Supplementary Data 2:

Description: The compiled data from 5 cores not providing semiquantitative results.
