# Supplementary material for: Measurements of heterogeneity in proteomics analysis of the nanoparticle protein corona across core facilities
Source: Nat Commun. 2022 Nov 3;13:6610. doi: 10.1038/s41467-022-34438-8 (PMC9633814; doi:10.1038/s41467-022-34438-8)
Supplement: Supplementary file 6 — Reporting Summary [file 41467_2022_34438_MOESM6_ESM.pdf]

nature portfolio

Corresponding author(s): Amir Ata Saei and Morteza Mahmoudi

Last updated by author(s): Oct 13, 2022

Reporting Summary

Nature Portfolio wishes to improve the reproducibility of the work that we publish. This form provides structure for consistency and transparency in reporting. For further information on Nature Portfolio policies, see our Editorial Policies and the Editorial Policy Checklist.

Statistics

For all statistical analyses, confirm that the following items are present in the figure legend, table legend, main text, or Methods section.

n/a	Confirmed	
☐	☒	The exact sample size (*n*) for each experimental group/condition, given as a discrete number and unit of measurement
☐	☒	A statement on whether measurements were taken from distinct samples or whether the same sample was measured repeatedly
☐	☒	The statistical test(s) used AND whether they are one- or two-sided<br>*Only common tests should be described solely by name; describe more complex techniques in the Methods section.*
☒	☐	A description of all covariates tested
☒	☐	A description of any assumptions or corrections, such as tests of normality and adjustment for multiple comparisons
☐	☒	A full description of the statistical parameters including central tendency (e.g. means) or other basic estimates (e.g. regression coefficient) AND variation (e.g. standard deviation) or associated estimates of uncertainty (e.g. confidence intervals)
☐	☒	For null hypothesis testing, the test statistic (e.g. *F*, *t*, *r*) with confidence intervals, effect sizes, degrees of freedom and *P* value noted<br>*Give P values as exact values whenever suitable.*
☒	☐	For Bayesian analysis, information on the choice of priors and Markov chain Monte Carlo settings
☐	☒	For hierarchical and complex designs, identification of the appropriate level for tests and full reporting of outcomes
☐	☒	Estimates of effect sizes (e.g. Cohen's *d*, Pearson's *r*), indicating how they were calculated

Our web collection on statistics for biologists contains articles on many of the points above.

Software and code

Policy information about availability of computer code

Data collection	Different core facilities used different software including Scaffold v.4.11.1, v.5.1, and v5.0.1, Proteome Discoverer 2.4, 2.4.0.305 and P2.2.0.388, PEAKS-XPro server, Peaks Studio 8.5, PEAKS Studio 10plus, Byonic v4.2.4, Sequest, MSFragger, Mascot 2.8 and Mascot Distiller v2.7.0 for data extraction (collection).
Data analysis	Data analysis was performed using R project version 3.6.1.

For manuscripts utilizing custom algorithms or software that are central to the research but not yet described in published literature, software must be made available to editors and reviewers. We strongly encourage code deposition in a community repository (e.g. GitHub). See the Nature Portfolio guidelines for submitting code & software for further information.

Data

Policy information about availability of data

All manuscripts must include a data availability statement. This statement should provide the following information, where applicable:

- Accession codes, unique identifiers, or web links for publicly available datasets
- A description of any restrictions on data availability
- For clinical datasets or third party data, please ensure that the statement adheres to our policy

Due to the blinding of core facility names in the current study, and since the .raw files can be traced back to the cores, the .raw data and associated individual data files are available upon request from corresponding authors. The extracted protein abundance data and relevant outputs of data analysis are provided in the Supplementary Data cited in the text. Supplementary Data 1 was used to generate Figs. 3-5 and Supplementary Figs. 4-5 (Supplementary Data 1 is the Source Data

for all the figures related to proteomics data). Different core facilities used the following databases including SwissProt TaxID 9606 downloaded on v.2017/05/10 with 42,153 entries, Swissprot database downloaded on 2021/02, UniProt human database (UP000005640) downloaded on 12/11/2020, UniProt downloaded on 07/02/2019, UniProt-Human database updated on 03/08/2021 with 20,379 entries, UniProt-human_20210508 database with 77,027 entries, UniProt (UP000005640) downloaded on 03/30/2021 with 20,310 protein entries, UniProt-homo_sapiens_20190201.fasta with 147,857 entries, NCBI and NcbiAV TaxID=9606 downloaded on v2017/10/30.

Human research participants

Policy information about studies involving human research participants and Sex and Gender in Research.

Reporting on sex and gender	NA
Population characteristics	NA
Recruitment	NA
Ethics oversight	NA

Note that full information on the approval of the study protocol must also be provided in the manuscript.

Field-specific reporting

Please select the one below that is the best fit for your research. If you are not sure, read the appropriate sections before making your selection.

[x] Life sciences [ ] Behavioural & social sciences [ ] Ecological, evolutionary & environmental sciences

For a reference copy of the document with all sections, see nature.com/documents/nr-reporting-summary-flat.pdf

Life sciences study design

All studies must disclose on these points even when the disclosure is negative.

Sample size	No statistical methods were used to predetermine sample size. A minimum of 3 individual measurements for all nanoparticle-related characterizations was performed to enable robust statistics. Appropriate statistical power was gained from at least 3 technical replicates for proteomics measurements and sample sizes were chosen based on similar studies published before.
Data exclusions	No data exclusion was done. However, non-semiquantitative data from 5 cores were not analyzed.
Replication	Each analysis/experiment was repeated 3 times and the average values were reported. All attempts at replication were successful.
Randomization	This is not relevant in the current study, as our study utilizes technical replicates of pooled plasma samples, with no covariates such as patient to patient variability.
Blinding	All 17 proteomics cores were numbered from 1 to 17 (for blinding) in all analyses described in the paper. Blinding of the investigators was not performed, as each core facility received an aliquot of the same sample (investigators were blinded in the sense that they were not informed that the other aliquots will be analyzed by other centers and that the collected data will be compared among the proteomics centers).

Reporting for specific materials, systems and methods

We require information from authors about some types of materials, experimental systems and methods used in many studies. Here, indicate whether each material, system or method listed is relevant to your study. If you are not sure if a list item applies to your research, read the appropriate section before selecting a response.

Materials & experimental systems

n/a	Involved in the study	
[x]	[ ]	Antibodies
[x]	[ ]	Eukaryotic cell lines
[x]	[ ]	Palaeontology and archaeology
[x]	[ ]	Animals and other organisms
[x]	[ ]	Clinical data
[x]	[ ]	Dual use research of concern

Methods

n/a	Involved in the study	
[x]	[ ]	ChIP-seq
[x]	[ ]	Flow cytometry
[x]	[ ]	MRI-based neuroimaging
